# Is paliperidone palmitate more effective than other long-acting injectable antipsychotics?

**DOI:** 10.1017/S0033291717003051

**Published:** 2017-10-17

**Authors:** R. Patel, E. Chesney, M. Taylor, D. Taylor, P. McGuire

**Affiliations:** 1 Department of Psychosis Studies, King's College London, Institute of Psychiatry, Psychology & Neuroscience, Box PO 63, De Crespigny Park, Denmark Hill, London, UK; 2 Pharmacy Department, South London and Maudsley NHS Foundation Trust, Denmark Hill, London, UK; 3 King's College London, Institute of Pharmaceutical Science, 5th Floor, Franklin-Wilkins Building, 150 Stamford Street, London, UK

**Keywords:** LAI, depot, paliperidone, xeplion, trevicta, schizophrenia, psychosis

## Abstract

**Background:**

Paliperidone palmitate is one of the most widely prescribed long-acting injectable (LAI) antipsychotics in the UK. However, it is relatively expensive and there are few data comparing its effectiveness to that of other LAI antipsychotics. We sought to address this issue by analyzing a large anonymized electronic health record (EHR) dataset from patients treated with LAI antipsychotics.

**Methods:**

EHR data were obtained from 1281 patients in the South London and Maudsley NHS Foundation Trust (SLaM) who started treatment with a LAI antipsychotic between 1 April 2011 and 31 January 2015. The number of days spent as a psychiatric inpatient and the number of admissions to a psychiatric hospital were analyzed in each of the 3 years before and after LAI prescription.

**Results:**

Patients treated with paliperidone palmitate (*n* = 430; 33.6%) had a greater number of inpatient days and a greater number of admissions in the year prior to treatment than those treated with other LAI antipsychotics. Nevertheless, in the 3 years after initiation there were no significant differences between paliperidone and the other LAI antipsychotics in the number of days as an inpatient (B coefficient 5.4 days, 95% confidence interval (CI) −57.3 to 68.2, *p* = 0.86) or number of hospital admissions (Incidence rate ratio 1.07, 95% CI 0.62 to 1.83, *p* = 0.82).

**Conclusion:**

Paliperidone palmitate was more likely to be prescribed in patients with more frequent and lengthy hospital admissions prior to initiation. However, the absence of differences in outcomes after initiation indicates that paliperidone palmitate was not more effective than other cheaper LAI antipsychotics.

## Introduction

One of the key factors that limits the clinical effectiveness of treatment of psychotic disorders with oral antipsychotic medication is poor adherence. Although long-acting injectable (LAI) antipsychotics may provide a means of overcoming this problem (Tiihonen *et al.* 2011; Marcus *et al.* 2015), a minority of patients prescribed antipsychotics (typically about 12% of the population investigated in the present study) (Pinto *et al.* 2010) are treated with LAIs. This may reflect patients being less willing to be treated via an intramuscular injection, and concerns about stigmatization, adverse effects and limiting patient autonomy (Johnson, 2009), concerns that may be shared by the prescribing clinician. In practice, LAIs are typically used in patients that require chronic treatment, who have a history of poor treatment adherence, frequent relapses or hospital admissions. Until relatively recently, the use of LAIs was in decline, but the advent of second-generation LAIs, which may have fewer adverse effects, has led to calls for their more widespread use (Barnes, 2005; Brissos *et al.* 2014; Bosanac & Castle, 2015).

A meta-analysis of randomized controlled trials (RCTs) comparing oral and LAI antipsychotics found no difference in risk of relapse in patients with schizophrenia (Kishimoto *et al.* 2014). On the other hand, a meta-analysis of mirror-image studies, where periods of oral and LAI antipsychotic in the same patients are compared, showed that LAIs reduced the relative risk of hospitalization by half (Kishimoto *et al.* 2013). These apparently conflicting findings may reflect an effect of study design that is particular to LAIs (Kirson *et al.* 2013). A review comparing different study designs to evaluate outcomes associated with LAIs (Haddad *et al.* 2015) found that two recent RCTs, which had a pragmatic design (Alphs *et al.* 2015; Schreiner *et al.* 2015) demonstrated better outcomes with paliperidone palmitate compared with oral antipsychotics. The main advantage of treatment with LAIs as opposed to oral antipsychotics is improved treatment adherence, but this benefit may be diminished in controlled trials: when a patient is taking part in a trial they may be more likely to take oral medication than in routine clinical care, and their adherence in a trial will usually be more closely monitored.

Second-generation antipsychotics may have fewer adverse effects than first-generation antipsychotics (Leucht *et al.* 2009), which may lead to the improved adherence, and a reduced risk of relapse and hospital admission (Leucht *et al.* 2011). At present, there are four second-generation LAIs licensed for clinical use: risperidone, olanzapine pamoate or embonate, aripiprazole and paliperidone palmitate. Paliperidone palmitate was introduced in the UK in 2011 and is the LAI formulation of oral paliperidone. In 2015, 5.0% of all community LAI prescriptions in the UK were for paliperidone palmitate, though due to its high cost it accounted for 25.1% of all spending on LAI antipsychotics (Health and Social Care Information Centre, 2005). This has led to an ongoing debate about the cost-effectiveness of treatment with the drug. In comparison with placebo, paliperidone is effective at reducing psychotic symptoms(Kramer *et al.* 2010; Alphs *et al.* 2011) and the risk of relapse and hospitalization (Hough *et al.* 2010; Kozma *et al.* 2011; Berwaerts *et al.* 2015), and is generally well tolerated (Coppola *et al.* 2012). In comparison with seven different oral antipsychotics in a single open-label trial, paliperidone palmitate was associated with a lower rate of treatment failure (40% *v.* 54%) at 15 months (Alphs *et al.* 2015). The only comparison between oral paliperidone and injectable paliperidone palmitate was made by comparing the results of two separate, but similarly designed placebo-controlled trials (Kramer *et al.* 2007; Hough *et al.* 2010). Here the injectable preparation was associated with a significantly lower risk of relapse (Markowitz *et al.* 2013).

To date, data comparing the efficacy of paliperidone palmitate with that of other LAIs in head to head clinical trials is sparse. In a study of 311 participants followed up for up to 24 months (which was not sponsored by the manufacturer), the efficacy of paliperidone palmitate was found to be no different to haloperidol decanoate (McEvoy *et al.* 2014). Compared to risperidone LAI, two studies (sponsored by the manufacturers of paliperidone palmitate) with 13 weeks of follow-up of 452 and 1220 participants, respectively found that paliperidone palmitate was non-inferior (Li *et al.* 2011; Pandina *et al.* 2011), but another study of 749 acutely symptomatic patients followed up for 53 weeks reported that it was less effective (Fleischhacker *et al.* 2012). In a study (sponsored by the manufacturers of aripiprazole LAI) of 295 participants followed up for 28 weeks, paliperidone palmitate has been shown to be inferior compared to aripiprazole LAI (Naber *et al.* 2015). Together, these studies suggest that paliperidone is effective, but not necessarily more effective than another second-generation LAIs. This limited evidence from clinical trials seems unlikely to account for the increasing popularity of the drug among prescribers. We sought to assess the effectiveness of paliperidone relative to other LAIs in a large sample that is more representative of the population of patients that are seen in clinical practice than those recruited to controlled treatment trials. We also conducted an audit of senior psychiatrists within the same mental healthcare service to better understand the rationale for choosing to prescribe paliperidone palmitate.

## Method

### Study setting and participants

The study was conducted using clinical data collected from patients receiving mental healthcare from the South London and Maudsley NHS Foundation Trust (SLaM). SLaM provides inpatient and community services for a catchment population of around 1.5 million people living in southeast London. We included patients aged between 16 and 65 years who had been started on a LAI between 1 April 2011 and 31 January 2015. This time period was chosen because paliperidone palmitate first became locally available on 1 April 2011. LAI medications were defined as any LAI antipsychotic listed in chapter 4.2.2 of the British National Formulary between 1 April 2011 and 31 January 2015. These were aripiprazole, flupentixol decanoate, fluphenazine decanoate, haloperidol, olanzapine embonate, paliperidone palmitate, pipotiazine palmitate, risperidone and zuclopenthixol decanoate (but not zuclopenthixol acetate). Using these criteria, data from 1281 people were available for analysis.

### Source of clinical data

Clinical data were obtained from the SLaM Biomedical Research Centre (BRC) Case Register, which contains anonymized electronic health records (EHRs) of over 270 000 patients. The clinical information documented includes structured fields (for demographic information) and de-identified unstructured free text fields from case notes and correspondence. Data were obtained from structured and unstructured clinical records using the Clinical Record Interactive Search tool (CRIS). CRIS is a bespoke database search and assembly tool that has supported a range of studies using clinical data from the SLaM BRC Case Register (Patel *et al.* 2015*a*, 2015*b*, 2015*c*, 2015*d*, 2016*a*, 2016*b*).

### Ethical approval

The SLaM BRC Case Register and CRIS have received ethical approval from the Oxfordshire Research Ethics Committee C (08/H0606/71+5) as an anonymized dataset for mental health research. A patient-led oversight committee provides governance for all projects conducted using these data. Any researcher wishing to use CRIS for a research study must undergo a rigorous approval procedure in accordance with UK Department of Health standards. A robust firewall and data security framework governs access to clinical data from the case register and only approved researchers are permitted to access data from the case register.

### Exposure

The exposure was defined as the first antipsychotic LAI medication prescribed to each patient included in the study. Clinical outcomes were measured after an index date defined as the date of the first prescription of the LAI plus 1 month. This definition was chosen to allow for adequate time for peak plasma levels of antipsychotic to be reached and to ensure that the exposure always occurred prior to measurement of clinical outcome measures.

### Clinical outcome measures

The primary outcome measure was the number of days spent as an inpatient in a psychiatric hospital in each of the three years before and after the index date. This was chosen because it represents an important measure of the burden of illness for individual patients, their family and carers, and mental healthcare services. It is also a key factor that determines the economic cost of mental health care (McCrone *et al.* 2008). The secondary outcome measure was the number of admissions to a psychiatric hospital in SLaM in each of the three years before and after the index date. Outcome data were collected up to 28 February 2015. Of the 1281 patients in the study, 980 had outcome data available at 1 year (i.e. index date prior to 28 February 2014), 623 at 2 years (i.e. index date prior to 28 January 2013) and 268 at 3 years (i.e. index date prior to 28 January 2012).

### Covariates

The following variables were extracted as categorical covariates for multivariable analyses: age, gender, ethnicity, marital status, diagnosis, borough of residence and whether started on LAI as an inpatient. The number of hospital admissions in the 3 years prior to the index date was extracted as a continuous covariate as a measure of illness severity. All categorical covariate data obtained were those recorded closest to the index date. Ethnicity was recorded according to categories defined by the UK Office for National Statistics. Marital status was recorded in the following categories: married or cohabiting; divorced or separated; single; unknown. Diagnosis of a psychotic disorder was defined according to ICD-10 and included schizophrenia or related disorders [schizophrenia (F20), delusional disorder (F22), schizophrenia-like disorders (F23, F28, F29)], schizoaffective disorder (F25), mania or bipolar disorder (F30, F31), psychotic depression (F32.3, F33.3), drug-induced psychosis (F1x.5) and any other psychotic disorder not otherwise specified.

### Statistical analysis

Stata (version 12.0) was used to analyze the data. Descriptive statistics for the exposure, outcome and covariate variables were obtained as frequencies and percentages for categorical variables and means and standard deviations for continuous variables. The association of starting paliperidone palmitate *v.* other antipsychotic LAIs was tested in the following analyses:

(i) Demographic and clinical covariates using multivariable binary logistic regression;
(ii) Number of days spent in hospital using multiple linear regression;
(iii) Number of hospital admissions using multivariable negative binomial regression.

Where missing data were present in covariate data (58 patients with no known marital status), the missing data category was included as a predictor variable in regression analyses.

### Audit of prescribers

A survey of Consultant Psychiatrists practising in SLaM was conducted to assess their views on LAI antipsychotic prescribing. The psychiatrists were invited to complete the survey which asked three questions:

(i) Which LAI antipsychotics do you prescribe regularly?
(ii) Which LAI antipsychotic would you choose to receive yourself?
(iii) If paliperidone LAI were no longer available to prescribe, would this be a good/neutral/bad thing?

The psychiatrists were also invited to provide free text comments on their responses to the questions.

## Results

### LAI antipsychotic exposure

Table 1 shows the breakdown of the different LAIs that were prescribed. Paliperidone palmitate was the most frequently prescribed LAI and 430 patients (33.6%) were treated with this drug. Compared with other LAIs, patients treated with paliperidone palmitate were more likely to have started treatment in hospital than in outpatient services, and were more likely to be female (Table 2).

**Table 1.** Prevalence of Antipsychotic LAI prescribing (n = 1281).

Antipsychotic LAI	Number in sample	Percentage (%)
Paliperidone palmitate	430	33.6
Zuclopenthixol decanoate	226	17.6
Flupentixol decanoate	203	15.9
Risperidone	160	12.5
Pipotiazine palmitate	114	8.9
Haloperidol	71	5.5
Fluphenazine decanoate	36	2.8
Aripiprazole	27	2.1
Olanzapine embonate	14	1.1

**Table 2.** *Binary logistic regression analysis of factors associated with starting paliperidone palmitate compared with other antipsychotic LAI, n* = *1281*

Factor	Group	Number in sample	Percentage started on paliperidone palmitate (%)	Unadjusted odds ratio	95% confidence interval, *p* value	^a^Adjusted odds ratio	95% confidence interval, *p* value
Age	Age 16–25 years	243	33.7	0.95	0.67–1.34, *p* = 0.75	0.99	0.68–1.45, *p* = 0.97
	26–35	337	35.0	Reference		Reference	
	36–45	301	30.9	0.83	0.60–1.16, *p* = 0.27	0.79	0.55–1.13, *p* = 0.20
	46–55	280	36.4	1.06	0.76–1.48, *p* = 0.72	0.98	0.69–1.41, *p* = 0.92
	56–65	120	29.2	0.76	0.49–1.20, *p* = 0.24	0.71	0.43–1.19, *p* = 0.20
Gender	Female	513	37.6	1.35	1.07–1.71, *p* = 0.01	1.37	1.04–1.79, *p* = 0.02
	Male	768	30.9	Reference		Reference	
Ethnicity	White	501	28.9	0.67	0.52–0.86, *p* = 0.002	1.06	0.79–1.41, *p* = 0.71
	Asian	71	33.8	0.84	0.50–1.41, *p* = 0.51	1.27	0.72–2.23, *p* = 0.41
	Black	597	37.9	Reference		Reference	
	Other ethnic group	112	31.3	0.75	0.48–1.15, *p* = 0.18	1.04	0.64–1.67, *p* = 0.88
Marital status	Married/cohabiting	129	41.1	1.38	0.95–2.01, *p* = 0.09	1.34	0.89–2.03, *p* = 0.16
	Divorced/separated	121	38.8	1.26	0.85–1.86, *p* = 0.25	1.19	0.77–1.84, *p* = 0.43
	Single	960	33.5	Reference		Reference	
	Widowed	13	7.7	0.17	0.02–1.28, *p* = 0.08	0.19	0.02–1.59, *p* = 0.13
	Marital status unknown	58	12.1	0.27	0.12–0.61, *p* = 0.001	0.73	0.31–1.75, *p* = 0.48
Diagnosis	Schizophrenia and related	743	37.8	Reference		Reference	
	Schizoaffective	77	40.3	1.11	0.69–1.79, *p* = 0.68	0.89	0.54–1.49, *p* = 0.67
	Bipolar disorder	27	44.4	1.32	0.61–2.85, *p* = 0.49	1.13	0.49–2.62, *p* = 0.78
	Psychotic Depression	84	26.2	0.58	0.35–0.97, *p* = 0.04	0.61	0.35–1.04, *p* = 0.07
	Drug-induced psychosis	15	53.3	1.88	0.67–5.24, *p* = 0.23	1.88	0.63–5.62, *p* = 0.26
	Other psychosis	335	22.7	0.48	0.36–0.65, *p* < 0.001	0.54	0.39–0.74, *p* < 0.001
Location	Inpatient	694	42.9	Reference		Reference	
	Outpatient	587	22.5	0.39	0.30–0.49, *p* < 0001	0.45	0.34–0.60, *p* < 0.001

a Adjusted for age, gender, ethnicity, marital status, diagnosis, location of starting LAI, number of admissions in the 3 years prior to starting LAI, and borough of residence.

### Clinical outcomes

**Fig. 1.**
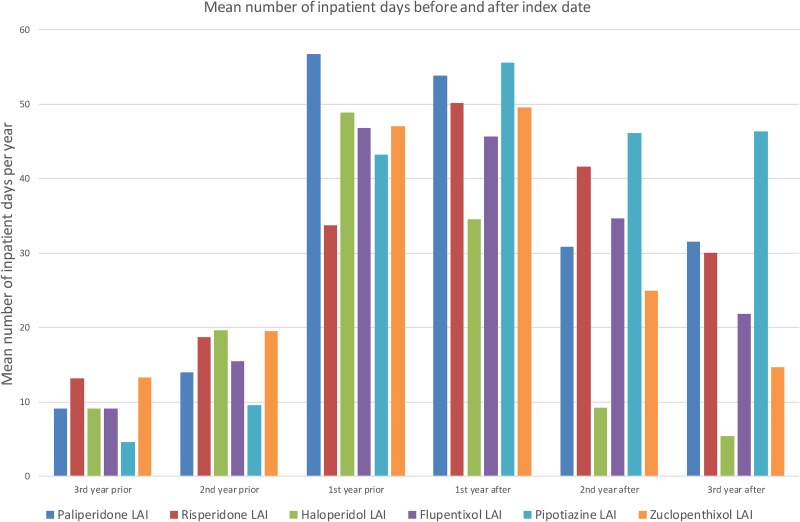
Mean number of days spent in a psychiatric hospital before and after starting Antipsychotic LAI.

**Fig. 2.**
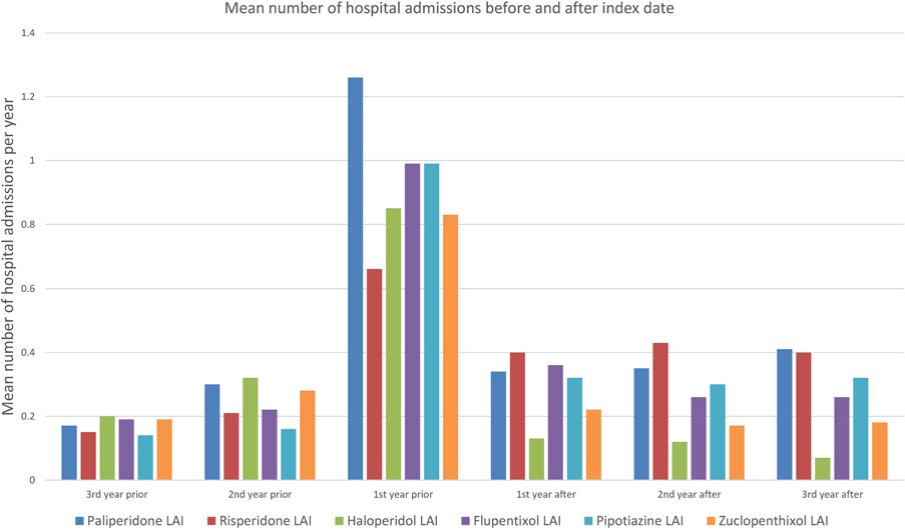
Mean number of admissions to a psychiatric hospital before and after starting Antipsychotic LAI.

Figure 1 shows the mean number of days spent in a psychiatric hospital, and Fig. 2 the mean number of admissions to a psychiatric hospital in the 3 years before and after the index date for each antipsychotic LAI, where data of more than 10 patients were available. Full data are presented in the Supplementary Material: eTables 1 and 2. In the year prior to the index date, patients prescribed paliperidone palmitate (*n* = 430) had a significantly greater number of inpatient days (*β* coefficient 12.3 days, 95% confidence interval (CI) 2.3 to 19.2, *p* = 0.001) and greater number of admissions (IRR 1.44, 95% CI 1.29 to 1.61, *p* < 0.001) compared with patients prescribed other antipsychotic LAIs (*n* = 851). However, after the index date, the association between paliperidone palmitate and the number of inpatient days or hospital admissions was no longer significant, compared with other antipsychotic LAIs (Table 3).

**Table 3.** Association of paliperidone palmitate v. other antipsychotic depots with clinical outcomes

Follow-up period (*n*)	Number of inpatient days		Number of hospital admissions	
	Unadjusted B coefficient (95% confidence interval, *p* value)	^a^Adjusted B coefficient (95% confidence interval, *p* value)	Unadjusted incidence rate ratio (95% confidence interval, *p* value)	^a^Adjusted incidence rate ratio (95% confidence interval, *p* value)
12 months (*n* = 980)	4.6 days (−7.6 to 16.8, *p* = 0.46)	−0.2 days (−12.3 to 11.9, *p* = 0.97)	1.19 (0.90–1.57, *p* = 0.22)	1.18 (0.88–1.58, *p* = 0.26)
24 months (*n* = 623)	1.5 days (−25.3 to 28.2, *p* = 0.92)	0.82 days (−27.3 to 28.9, *p* = 0.95)	1.14 (0.86–1.51, *p* = 0.36)	1.00 (0.73–1.35, *p* = 0.98)
36 months (*n* = 268)	25.9 days (−41.9 to 93.7, *p* = 0.45)	5.4 days (−57.3 to 68.2, *p* = 0.86)	1.33 (0.80–2.19, *p* = 0.27)	1.07 (0.62–1.83, *p* = 0.82)

a Adjusted for age, gender, ethnicity, marital status, diagnosis, borough of residence, whether started on LAI as an inpatient and number of hospital admissions in the 3 years prior to the index date.

### Audit of prescribers

Around 80 psychiatrists providing care to patients with schizophrenia in the community and in psychiatric hospitals were invited to respond to the survey. Thirty-one responses were received. The most frequently prescribed LAIs (Supplementary Material: eFig. 1) were paliperidone palmitate (87.1%), zuclopenthixol decanoate (80.6%) and flupentixol decanoate (58.1%). In response to which LAI the psychiatrists would choose to receive themselves (eFig. 2), paliperidone palmitate (25.0%) and aripiprazole (32.1%) were the most popular. Twenty-three out of 31 psychiatrists (74.2%) felt that if paliperidone LAI were no longer available to prescribe, this would this be a bad thing. Free text comments (Supplementary Material) highlighted concerns regarding side effects of first-generation antipsychotic LAIs such as haloperidol, ease of administration of paliperidone palmitate via the deltoid route, and monthly (rather than 2 weekly) frequency of administration as a benefit of paliperidone palmitate.

## Discussion

Since its launch in 2011, paliperidone palmitate has rapidly become one the most frequently prescribed LAI antipsychotics for the treatment of psychotic disorders, despite a lack of clear evidence from clinical trials that it is more effective than existing LAI antipsychotics. The aim of the present study was to compare its effectiveness to other LAIs in a large clinical sample of patients. Our main findings were that paliperidone palmitate was more likely to be used in patients who had relatively high numbers of hospital admissions and inpatient days in the previous year, and who had been inpatients when the treatment was started. One explanation for the greater proportion of patients starting paliperidone palmitate as an inpatient could be that inpatient admission reflects a recent deterioration in mental state (possibly due to poor oral medication adherence) which leads to clinicians starting a LAI antipsychotic (Ascher-Svanum *et al.* 2009).

In contrast, there was no association with increased admissions and inpatient days in the 3 years after starting paliperidone palmitate. This could be explained by three possible mechanisms. First, because the patients who were prescribed paliperidone palmitate had features associated with a relatively poor prognosis, paliperidone palmitate might be more effective than other LAI antipsychotics. However, another possibility is that the severity of illness in the sample regresses toward the mean, such that patients who were ill enough to require the prescription of a new LAI would in following years be less likely to require hospitalization. As those prescribed paliperidone palmitate had the most inpatient days before admission, they might therefore require relatively less hospitalization in subsequent years. Finally, there is evidence that the acute effect of oral antipsychotics is greatest in patients who are severely unwell (Furukawa *et al.* 2015), so the improvement seen with paliperidone may be related to the severity of illness in these patients, rather than the effect of paliperidone *per se*.

Data from four RCTs studying over 3000 patients do not suggest that the efficacy of paliperidone palmitate is greater than that of other LAIs (Li *et al.* 2011; Pandina *et al.* 2011; Fleischhacker *et al.* 2012; McEvoy *et al.* 2014). Of the three trials comparing paliperidone palmitate with risperidone LAI, one found paliperidone palmitate to be inferior. However, this trial used a sub-optimal dosing regimen which did not achieve therapeutically effective plasma levels of paliperidone (Fleischhacker *et al.* 2012). However, a study comparing paliperidone palmitate with aripiprazole once-monthly LAI found that aripiprazole was associated with better health-related quality of life and reduced rates of discontinuation compared to paliperidone palmitate (Naber *et al.* 2015). The clinical utility of antipsychotic LAIs is not solely a function of their effectiveness: it is also dependent on their adverse effects and how well patients tolerate the treatment. The survey of clinicians from the same mental health service indicated that they are aware of the relatively high cost of paliperidone palmitate, but prescribe it because they perceive it as being better tolerated by patients than other LAI antipsychotics. This is supported by recent findings indicating that discontinuation rates of paliperidone LAI are lower than the LAI preparations of risperidone, haloperidol and olanzapine (Decuypere *et al.* 2017). Furthermore, a 3-monthly preparation of paliperidone palmitate has recently been introduced and been shown to be equivalent in efficacy to the once-monthly preparation (Savitz *et al.* 2016). The reduced frequency of administration of the 3-monthly preparation may improve tolerability and could be an important factor in determining which LAI antipsychotic to prescribe.

With respect to effectiveness, the results of the present study are consistent with those from previous observational studies. In patients who switched from LAI risperidone to either paliperidone palmitate or another oral antipsychotic (Voss *et al.* 2015), the risk of relapse was lower [HR 0.54 (CI 0.32–0.92)] in those who switched to paliperidone. In another study, new prescription of paliperidone palmitate was associated with a reduction in admission and inpatients days compared with previous years (Taylor & Olofinjana, 2014). Decisions about the choice of LAI antipsychotic treatment may also involve an evaluation of cost-effectiveness. This is a particular issue with paliperidone palmitate in view of its high cost relative to other LAIs (Healthand Social Care Information Centre, 2005). Despite this, health economic studies, sponsored by the manufacturer, suggest that the additional cost of paliperidone palmitate compared to other antipsychotic treatments is outweighed by savings in healthcare and criminal justice system costs (Mehnert *et al.* 2012; Zeidler *et al.* 2013; Muser *et al.* 2015).

The main strength of this study is its large sample size and its generalizability to clinical practice. Capturing data from over 1200 patients prescribed LAIs, it is well powered and allows for comparison between most commonly prescribed LAI antipsychotics. We were able to compare outcomes between nine LAI antipsychotics approved by the British National Formulary, with data from over 100 patients for five of them, which would not be feasible in a conventional interventional study. Furthermore, we obtained data on clinicians’ perspectives with respect to antipsychotic LAI prescribing which provided a unique insight into perceptions of efficacy and tolerability, which would not have been possible to obtain from EHR data alone.

The data in the present study were not controlled, as they would be in a randomized trial. On the other hand, the sample was more representative of the population of patients that are seen in routine clinical practice that would have been the case in a typical clinical trial, the sample size was larger, and duration of follow-up was longer. However, it should be noted that although the follow-up period in the present study was up to 3 years, rates of discontinuation for paliperidone palmitate have been shown to be 35% after 1 year(Attard *et al.* 2014) and up to 84% for risperidone LAI after 3 years (Taylor *et al.* 2009). For this reason, a number of patients may have switched to an alternative antipsychotic or stopped receiving any antipsychotic therapy during the follow-up period. A further limitation was the lack of data on psychotropic prescribing prior to initiating a LAI antipsychotic as these data were not comprehensively documented in the EHR. The failure of previous antipsychotic therapy due to poor efficacy or poor treatment adherence may have explained the greater number of inpatient days and hospital admissions observed in patients prior to starting paliperidone palmitate.

In conclusion, this study suggests that paliperidone palmitate was at least as effective as other LAI antipsychotics. A key issue to address in future studies is whether paliperidone is more effective than other LAIs when given to patients who are matched for illness severity and prognosis.

## Supplementary material

For supplementary material accompanying this paper visit https://doi.org/10.1017/S0033291717003051. click here to view supplementary material

## Data sharing

No additional data are available.
