# Supplementary material for: Is paliperidone palmitate more effective than other long-acting injectable antipsychotics?
Source: Psychol Med. 2017 Oct 17;48(10):1616–23. doi: 10.1017/S0033291717003051 (PMC6088783; doi:10.1017/S0033291717003051)
Supplement: Supplementary file 1 [file S0033291717003051sup001.docx]

**Title**

Is Paliperidone Palmitate more effective than other long-acting injectable antipsychotics?

**Authors**

Rashmi Patel BM BCh PhD^1^, Edward Chesney BM BCh^1^, Matthew Taylor BM BCh DPhil^1^, David Taylor PhD^2,3^ and Philip McGuire MD PhD^1^

**Author affiliations**

1. King’s College London, Department of Psychosis Studies, Institute of Psychiatry, Psychology & Neuroscience, Box PO 63, De Crespigny Park, Denmark Hill, London SE5 8AF, UK
2. South London and Maudsley NHS Foundation Trust, Pharmacy Department, Denmark Hill, London SE5 8AZ, UK
3. King’s College London, Institute of Pharmaceutical Science, 5th Floor, Franklin-Wilkins Building, 150 Stamford Street, London SE1 9NH, UK

# Supplementary Data

eTable 1

| **Mean number of days spent in a psychiatric hospital before and after the index date (n, std dev)** | | | | | | |
| --- | --- | --- | --- | --- | --- | --- |
| **Antipsychotic LAI** | **3rd year prior** | **2nd year prior** | **1st year prior** | **1st year after** | **2nd year after** | **3rd year after** |
| Aripiprazole | 10.7 (27, 26.5) | 20.6 (27, 32.7) | 48.7 (27, 46.7) | n=0 | n=0 | n=0 |
| Flupentixol Decanoate | 9.1 (203, 28.6) | 15.5 (203, 46.7) | 46.8, (203, 57.8) | 45.6 (159, 80.9) | 34.7 (106, 88.7) | 21.8 (54, 72.6) |
| Fluphenazine Decanoate | 4.8 (36, 16.6) | 2.7 (36, 10.5) | 28.4 (36, 47.4) | 43.4 (32, 98.1) | 48.6 (17, 89.0) | 11.1 (8, 26.6) |
| Haloperidol | 9.1 (71, 29.3) | 19.6 (71, 56.2) | 48.9 (71, 69.9) | 34.5 (45, 80.4) | 9.2 (25, 29.0) | 5.4 (14, 20.3) |
| Olanzapine Embonate | 17.6 (14, 43.5) | 29.7 (14, 86.6) | 112.6 (14, 134.3) | 98.1 (13, 123.3) | 52.3 (7, 138.3) | 183.0 (2, 258.8) |
| Paliperidone Palmitate | 9.1 (430, 31.1) | 14.0 (430, 35.2) | 56.7 (430, 53.4) | 53.9 (329, 96.5) | 30.9 (203, 76.8) | 31.6 (51, 70.1) |
| Pipotiazine Palmitate | 4.6 (114, 18.9) | 9.6 (114, 32.1) | 43.2 (114, 41.7) | 55.6 (94, 92.8) | 46.1 (57, 97.6) | 46.3 (38, 94.9) |
| Risperidone | 13.2 (160, 50.6) | 18.7 (160, 60.9) | 33.7 (160, 61.3) | 50.2 (141, 88.9) | 41.6 (106, 86.1) | 30.0 (62, 70.5) |
| Zuclopenthixol Decanoate | 13.3 (226, 47.6) | 19.5 (226, 57.5) | 47.0 (226, 69.6) | 49.6 (167, 92.4) | 25.0 (102, 76.4) | 14.7 (39, 61.0) |

eTable 2

| **Mean number of psychiatric hospital admissions before and after the index date (n, std dev)** | | | | | | |
| --- | --- | --- | --- | --- | --- | --- |
| **Antipsychotic LAI** | **3rd year prior** | **2nd year prior** | **1st year prior** | **1st year after** | **2nd year after** | **3rd year after** |
| Aripiprazole | 0.30 (27, 0.61) | 0.56 (27, 0.64) | 1.3 (27, 1.0) | n=0 | n=0 | n=0 |
| Flupentixol Decanoate | 0.19 (203, 0.51) | 0.22 (203, 0.56) | 0.99 (203, 1.02) | 0.36 (159, 0.79) | 0.26 (106, 0.71) | 0.26 (54, 0.83) |
| Fluphenazine Decanoate | 0.06 (36, 0.23) | 0.11 (36, 0.40) | 0.72 (36, 0.85) | 0.06 (32, 0.25) | 0.47 (17, 1.01) | 0.25 (8, 0.46) |
| Haloperidol | 0.20 (71, 0.58) | 0.32 (71, 0.77) | 0.85 (71, 0.90) | 0.13 (45, 0.55) | 0.12 (25, 0.44) | 0.07 (14, 0.27) |
| Olanzapine Embonate | 0.07 (14, 0.27) | 0.14 (14, 0.36) | 0.86 (14, 0.77) | 0.0 (13, 0.0) | 0.0 (7, 0.0) | 0.0 (2, 0.0) |
| Paliperidone Palmitate | 0.17 (430, 0.49) | 0.30 (430, 0.60) | 1.26 (430, 0.95) | 0.34 (329, 0.69) | 0.35 (203, 0.93) | 0.41 (51, 0.78) |
| Pipotiazine Palmitate | 0.14 (114, 0.46) | 0.16 (114, 0.41) | 0.99 (114, 0.99) | 0.32 (94, 0.66) | 0.30 (57, 0.60) | 0.32 (38, 0.66) |
| Risperidone | 0.15 (160, 0.45) | 0.21 (160, 0.48) | 0.66 (160, 0.88) | 0.40 (141, 0.71) | 0.43 (106, 1.00) | 0.40 (62, 0.90) |
| Zuclopenthixol Decanoate | 0.19 (226, 0.56) | 0.28 (226, 0.63) | 0.83 (226, 0.95) | 0.22 (167, 0.52) | 0.17 (102, 0.42) | 0.18 (39, 0.45) |

eFigure 1

eFigure 2

Survey comments about paliperidone palmitate

‘Some pts seem to tolerate it better if EPSEs an issue for haloperidol. Plus licence to give deltoid is attractive to pts. With haloperidol strictly the dosing regime is for deltoid loading only, then as I understand it as least, continuing deltoid long term isn't licensed?’

‘There are situations in which it is the best option in terms of efficacy and side effects. We are careful in choosing to use it as we do understand the significant cost implications. We are now using low dose haldol first line.’

‘Exceptionally low side effects found. Minimally invasive in a persons life - 12 per year. Can have in arm or back-side - overcoming dignity issues. Enable patients to get on with their lives. Also low level feelings of numbness with older depots - patients relate head fells clearer, as if weight lifted. Suspect much higher levels of compliance, reducing relapse, enabling discharge to GP, and lower cost in long run (i.e. our patients may need a few admissions to trial no meds, and therefore such benefits may take few years to emerge.’

‘Can be useful depot for some patients but quite a lot of patients don't do well on it.’

‘It seems to be best option for some who are burdened by side effects with other classes. Also patient choice in relation to site of injection.’

‘I feel it is more a drive lately than its actual efficacy or side effect profile.’

‘A number of our difficult to treat chronic service users have been far more stable on paliperidone depot compared to typical depots and have reduced OBD use by hundreds already. We will be presenting the data to David Taylor shortly.’

‘It has been shocking at how this drug has been dosed and marketed- I have seen quite a lot of TD in young people with it, and the data used to extol its virtues above drugs such as Flupenthixol, was poor.’

‘It has helped and is still helping many patients, it should definitely remain an option.’

‘Patients should be able to select from the widest range of effective options. Pharmacology only takes up a small proportion of the overall budget. It is less costly than psychological treatments which don't make much difference anyway.’
